# Tolerability and Safety of Miltefosine for the Treatment of Cutaneous Leishmaniasis

**DOI:** 10.3390/tropicalmed9090218

**Published:** 2024-09-19

**Authors:** Nadav Astman, Chen Arbel, Oren Katz, Aviv Barzilai, Michal Solomon, Eli Schwartz

**Affiliations:** 1 Department of Dermatology, Chaim Sheba Medical Center, Tel-Hashomer 5262000, Israel; 2 Israel Defense Force (IDF) Medical Corps, Tel-Hashomer, Israel; 3 Sackler Faculty of Medicine, Tel Aviv University, Tel Aviv 6997801, Israel; 4 Department of Dermatology, Tel Aviv Sourasky Medical Center, Tel Aviv 6423906, Israel; 5 Center for Geographic Medicine, Chaim Sheba Medical Center, Tel-Hashomer 5262000, Israel

**Keywords:** cutaneous leishmaniasis, miltefosine, tolerability, safety, treatment

## Abstract

Miltefosine, an orally administered drug, is an important component of the therapeutic arsenal against visceral and mucosal forms of leishmaniasis. However, data regarding the safety and tolerability of miltefosine treatment for cutaneous leishmaniasis (CL) are relatively limited. The aim of this study was to evaluate the tolerability, safety, and adverse events (AEs) of miltefosine treatment in patients with CL. In this cohort study, we reviewed the medical records of all miltefosine-treated patients between 1 January 2016 and 31 December 2022, at Israel Defense Forces military dermatology clinics and the dermatology and Tropical Medicine Clinics at Chaim Sheba Medical Center, Ramat-Gan, Israel. A total of 68 patients (54 males, 79%) with a median age of 30.3 ± 15.6 years (range: 18–88) were included in this study. *Leishmania* species were identified as *L. major* (n = 37, 54.4%), *L. tropica* (n = 12, 17.6%), *L. braziliensis* (n = 18, 26.5%), and *L. infantum* (n = 1, 1.5%) using polymerase chain reaction (PCR). Miltefosine tablets were administered orally at a dose of 50 mg, three times daily, for 28 days. Overall, 44 patients (65%) completed the 28-day treatment, and the remaining patients required dose reduction or early discontinuation of treatment. AEs (of any degree) were common, reported in 91% of patients. Both previously reported and previously unreported AEs were documented. Gastrointestinal symptoms (66.1%) and malaise (23.5%) typically occurred during the first two weeks of treatment and tended to subside. Other AEs, including acute renal failure (20.6%), sudden and severe pleuritic chest pain (7.6%), acne exacerbation (11.8%), suppuration of CL lesions (17.8%), and AEs related to the male genitourinary system (39.6% of males), typically occurred towards the end of treatment. The latter included testicular pain, epididymitis, diminution or complete absence of ejaculate, inability to orgasm, and impotence. Severe AEs necessitated treatment discontinuation (29.4%) or hospitalization (10.3%). URTI-like symptoms, arthritis, cutaneous eruption, pruritus, and laboratory abnormalities were also observed. Overall, the cure rate (for all patients combined) evaluated 3 months after the completion of treatment was 60%. The tolerability of miltefosine treatment for CL is low. Close clinical and laboratory monitoring is required during treatment, as severe AEs are not uncommon. As new insights regarding its toxicities emerge, further studies are required to define the role of miltefosine in the treatment of CL.

## 1. Introduction

Miltefosine is a phosphatidylcholine analog with anti-leishmanial activity achieved through multiple pathways [1,2,3]. Although the drug was initially used for the treatment of several solid malignancies, it was never routinely used for this indication because of its limited efficacy and poor tolerability [4,5,6]. Early in vitro and in vivo studies, followed by clinical trials, demonstrated its remarkable efficacy against visceral leishmaniasis (VL) caused by *L. donovani.* The high cure rate achieved with miltefosine and its great advantage of being taken orally were considered significant therapeutic breakthroughs and revolutionized the therapeutic approach for this fatal disease, especially in rural areas [1,7,8,9]. In 2002, miltefosine was granted Orphan Drug Status by the European Drug Agency and, in the same year, it was also registered for the treatment of VL in India [9]. In 2011, miltefosine was included on the World Health Organization’s essential medicines list. Later, in 2014, miltefosine was finally registered by the U.S. Food and Drug Administration (FDA) for the treatment of VL caused by *L. donovani* and for cutaneous leishmaniasis (CL) and mucosal leishmaniasis (ML) caused by members of the *L. viannia* subgenus (e.g., *L. braziliensis*, *L. guyanensis*, and *L. panamensis*) for subjects aged 12 years and above [9].

Miltefosine may cause fetal harm, fetal death, and teratogenicity and should be avoided during pregnancy. Because of its prolonged half-life in blood, females with reproductive potential should be advised to use effective contraception during therapy and for 5 months following the completion of therapy.

Despite early reports describing the poor tolerability of miltefosine as an antineoplastic drug, most studies in VL have reported surprisingly favorable tolerability, with AEs that were generally mild and transient [8,10,11,12]. However, data regarding the efficacy and safety of miltefosine for the treatment of New World and Old World CL are more limited and based on relatively small cohorts [13,14,15,16,17,18,19,20,21,22,23,24,25,26]. In these studies, miltefosine demonstrated cure rates of approximately 80% for Old World CL and lower for New World CL, ranging between 50 and 80%. The safety and tolerability observed in these studies were generally favorable. However, other later studies revealed clinically significant but previously unreported AEs, including male reproductive system disturbances and the exacerbation of gout. Similarly, the WHO has recently published a statement regarding a potential risk for serious ocular disorders, including blindness, in patients treated with miltefosine for post-kala-azar dermal leishmaniasis [22,27,28].

In this multicenter retrospective study, we describe the AEs, safety, and tolerability of miltefosine treatment prescribed for a series of 68 patients with CL and provide a literature review regarding this topic.

## 2. Methods

We reviewed the medical records of all miltefosine-treated patients diagnosed with CL at the Israel Defense Forces military dermatology clinics and the dermatology and Tropical Medicine Clinics at Chaim Sheba Medical Center, Ramat-Gan, Israel, between 1 January 2016 and 31 December 2022. Epidemiological data obtained from the medical records included age, sex, and weight. For the detection of *Leishmania* species, we used polymerase chain reaction combined with restriction fragment length polymorphism (PCR-RFLP). For the detection of *L. major, L. tropica*, and *L. infantum*, the target gene was ITS1, while for the detection of New World species, the target gene was HSP70 [29,30]. The potential benefits and risks of miltefosine were discussed with all patients prior to treatment initiation. In most cases, miltefosine was offered as a second-line treatment after the failure of previous local and/or systemic treatment with sodium stibogluconate, meglumine antimoniate, or liposomal amphotericin B. However, it was also used as a first-line treatment for patients who refused intravenous or intramuscular treatment. Given the modest rate of clinical improvement in CL lesions after antileishmanial treatment, we allowed for a period of 1 to 3 months (following previous intralesional or systemic treatment, respectively) before assessing clinical response and determining the need for second-line treatment with miltefosine. This relatively extended period allowed for clear differentiation between AEs that may be attributable to different treatments.

The patients were instructed to take miltefosine tablets orally at an initial dose of 50 mg, three times daily (TID), for 28 days. Based on the patients’ responses and physicians’ clinical judgments, treatment was prolonged in a few selected cases. In other cases, the development of significant AEs necessitated the early discontinuation of treatment or dose reduction. Clinical evaluation and laboratory tests, including complete blood count, serum electrolytes, C reactive protein (CRP), creatinine phosphokinase (CPK), kidney and liver function tests, and amylase and lipase, were performed at the initiation of miltefosine treatment and every 1–2 weeks thereafter. Pregnancy issues were discussed with all women of child-bearing age, and they were instructed to use effective contraceptives during treatment and 5 months thereafter. 

Early discontinuation was defined as the discontinuation of treatment due to an inability to complete the 28-day treatment course. Dose reduction was defined as dose modifications resulting from poor tolerability to miltefosine AEs. 

AEs were classified as mild (grade 1: self-limiting, requiring no clinical intervention), moderate (grade 2: requiring clinical intervention), or severe (grade 3: requiring the termination of treatment). The following clinical and laboratory parameters were defined as obligating criteria for treatment discontinuation: 1. Referral to emergency room or hospitalization that were related in high probability to miltefosine treatment. 2. A creatinine level of ≥1.5 mg/dL. 3. An increase in LFT X 2.5 of the upper normal limit. 4. Severe testicular pain interfering with daily life activities or sonographic findings consistent with epididymitis. This study was approved by the institutional review boards (IRBs) of the IDF (2130-2020) and Chaim Sheba Medical Center (7332-20).

### Statistical Analysis

In this study, the values are presented as the mean (standard deviation; SD) or number (percentage). Categorical variables were compared using an χ^2^ test or Fisher’s exact test, and continuous variables were tested using the Wilcoxon two-sample test. A *p*-value < 0.05 was considered statistically significant. All the analyses were performed using SPSS 24.0 (SPSS, Chicago, IL, USA).

## 3. Results

Our cohort study included 68 patients, 54 (79.4%) males and 14 (20.6%) females, who were treated with miltefosine for CL. The basic characteristics of patients and the causative leishmania species are presented in Table 1. Although the difference was not statistically significant, females were, on average, slightly older than males. The average age was 29.1 years (SD = 15.5) for males and 35 years (SD = 15.8) for females with a *p*-value of 0.34. Body weight, as expected, was higher among males (*p*-value = 0.0002). Old World species (*L. major*, *L. tropica*, and *L. infantum*) were acquired in endemic areas in Israel, while all New World species (including *L. braziliensis*) occurred among travelers who had returned from South American countries, mainly Bolivia and Brazil. Five male patients suffered mucosal lesions at presentation. Differences in causative *Leishmania* species between genders were not statistically significant (*p*-value = 0.21).

Miltefosine was prescribed as the first treatment for 31 patients (45.6%), while 37 patients (54.4%) received it after the failure of previous antileishmanial treatment, mostly topical. Overall, the cure rate (for all patients combined) evaluated 3 months after the completion of treatment was 60%.

Tolerability to miltefosine treatment is demonstrated in Table 2. Only 44 patients (65%) completed the 28-day miltefosine treatment course as prescribed; meanwhile, 20 patients (29.4%) prematurely discontinued the treatment because of significant AEs, an additional 4 (6%) required dose reduction or temporary treatment cessation, and 9 patients (13.2%) suffered severe AEs that necessitated referral to an emergency room or hospitalization.

Nearly all patients reported AEs (of any severity); only six patients (8.8%) were symptom-free during the 28-day treatment period (Table 2). Although not statistically significant, a higher proportion of male patients completed the 28-day treatment compared with female patients (67.9% vs. 53.3%), despite the similar proportion of patients suffering moderate or severe AEs in both genders (Table 2).

Overall, the occurrence of AEs and laboratory abnormalities among males and females was similar, with no significant difference found between groups (Table 3). In addition, no difference in tolerability or AE rates was found between those who used miltefosine as a first- or second-line treatment. A detailed description of AEs by physiological systems follows.

Gastrointestinal AEs (GI-AEs): GI AEs, including nausea or vomiting, abdominal pain, and diarrhea (at any combination), were the most common AEs, being reported by 66% of the subjects. GI AEs were most often reported during the first two weeks of treatment and were usually alleviated thereafter. Taking miltefosine postprandially usually improves these symptoms. 

**Cutaneous AEs:** Worsening of existing leishmanial cutaneous lesions, including increased erythema, swelling, local pain, and suppuration (with or without the appearance of subcutaneous nodules or regional lymphadenopathy), were observed in 12 cases (17.6%). The exacerbation or new appearance of acne and acneiform folliculitis were observed in eight patients (11.8%), all of whom were young soldiers. In one case, acne exacerbation was accompanied by the development of an inflamed pilonidal cyst.

One female patient developed a bullous eruption that appeared soon after treatment initiation, and another patient developed acute generalized pruritus, which appeared within an hour after the oral intake of the drug during the last 10 days of treatment. In both cases, cutaneous manifestations subsided shortly after the discontinuation of treatment. 

Musculoskeletal chest and back pain: Six patients (8.8%) experienced an episode of chest pain that was severe enough to require referral to an emergency room and/or hospitalization. Patients reported intense back or chest pain, exacerbated during deep breathing or movement, to a degree interfering with daily activities, and, in some cases, leading to an inability to stand or walk. Thorough medical investigation to exclude cardiac or pulmonary embolism, including electrocardiogram (ECG), chest X-ray, cardiac echocardiogram, chest CT scan (in some cases), and biochemical tests, including creatinine phosphor-kinase (CPK) and cardiac enzymes, were unremarkable. In all cases, the symptoms fully subsided after treatment discontinuation and did not recur.

AEs related to the male reproductive system: A total of 21 male patients (39% of male patients) reported one or more AEs related to the male reproductive system (Table 3). The reported AEs included testicular pain (n = 15 patients, 28.3%), decreased volume of ejaculation (n = 11 subjects, 20.8%), inability to orgasm, erectile dysfunction, and decreased libido (n = 5, 9.4% each). These AEs typically appeared during the third or fourth week of treatment and required, in some cases, analgesics or the discontinuation of treatment. Ultrasonography, performed for three subjects who suffered severe orchialgia, demonstrated findings consistent with epididymitis. Treatment with antibiotics and non-steroidal anti-inflammatory drugs was initiated, resulting in a complete resolution of the symptoms within days. 

Renal function impairment and acute renal failure: Elevated serum creatinine levels were documented in 14 patients (20.6%). These AEs typically occurred during the 3rd or 4th week of treatment. For six subjects, the creatinine level was only mildly elevated (≤1.4 mg/dL); however, eight subjects suffered acute renal failure with creatinine values ranging between 1.5 and 8.7 mg/dL. An interesting observation was that an increase in the CRP level usually preceded an increase in the creatinine level. 

Two young, healthy men who suffered CL caused by *L. braziliensis* developed arthritis during miltefosine treatment. The first presented on the 28th day of treatment with a swollen, tender knee. Sterile fluid was aspirated from his knee, and PCR for leishmania and bacterial culture were negative. Uric acid levels were normal. The second patient also presented towards the end of miltefosine treatment with generalized, disabling musculoskeletal pain. He was hospitalized, and a diagnosis of idiopathic unilateral seronegative sacroiliitis was established. In both cases, the arthritis rapidly resolved after discontinuing miltefosine and initiating treatment with non-steroidal anti-inflammatory drugs (NSAIDs). 

Additional laboratory abnormalities that were documented during miltefosine treatment are presented in Table 3, including increases in liver function tests (LFTs; 12 subjects, 17.6%), anemia (3 subjects, 4.4%), hyperkalemia (1 subject, 1.5%), and leukocytosis (1 subject, 1.5%). 

AEs leading to the discontinuation of treatment or dose modifications are listed as frequencies in Table 4. Overall, 24 subjects (35.3%) discontinued treatment prematurely or required dose modifications. Acute renal failure (for all) and orchialgia (for men) were the leading causes of treatment discontinuation. AEs leading to permanent discontinuation of treatment were equally prevalent among males and females, although fewer males required temporary discontinuation or dose modification because of AEs. Renal failure was equally prevalent between genders, while females tended to suffer more frequently from severe GI symptoms.

## 4. Discussion

The main findings of our study are as follows: (1). Tolerability to miltefosine treatment is low, as many patients failed to complete the treatment course as prescribed. (2). We not only expand the data regarding known AEs but also describe previously unreported AEs. (3). The toxicities of miltefosine may be significant and more frequent than previously reported. (4). Close clinical and laboratory monitoring is required throughout treatment, as severe AEs are not uncommon. (5). In all cases, the AEs observed in our cohort study fully resolved without residual sequelae.

The aim of our study was to further expand the knowledge regarding the safety and tolerability of miltefosine for cutaneous leishmaniasis, as data on this topic are relatively limited. A systematic review that describes AEs following different systemic treatments given for VL was recently published. That study revealed that the safety outcomes in many studies were poorly reported, with substantial missing information regarding the timing and frequency of AEs. Deaths specifically related to miltefosine in clinical trials—although rare—have also occurred but have not been described in detail nor mentioned frequently in the literature thereafter [31].

The tolerability of miltefosine treatment in our cohort was low, with only 65% of patients completing the 28-day treatment as prescribed. These results demonstrate lower tolerability and higher toxicity than might have been expected as, in some studies, miltefosine was considered well-tolerated by both adults and children [11,12,23]. In earlier studies evaluating miltefosine as an anti-cancer drug in patients with metastatic solid tumors, 54 patients received a daily dose of 100 mg, which was increased to 150 mg/d and further to 200 mg/d. In this study, 22% of patients could not tolerate a daily dose greater than 100 mg [6].

Variability in tolerability may be partially explained by differences in population characteristics. In this regard, it has been proposed that tolerability among children is more favorable because of the faster metabolism of the drug [32]. Nevertheless, tolerability may be influenced by the severity of the underlying disease (potentially fatal VL vs. self-limiting CL) and the availability of alternative treatments, as both may influence patients’ adherence to treatment and clinician’s considerations in the presence of evolving AEs.

Gastrointestinal AEs were the most common AEs associated with miltefosine. In many reports, GI AEs were mild and transient [8,10,12], while in others, GI AEs occurred in up to 97% of patients, with approximately one-third requiring antiemetic therapy [22,27]. In our study, GI AEs were reported by 66% of the patients, necessitating the early discontinuation of treatment for some. Most GI AEs were worse during the first 2 weeks of treatment and tended to subside—both in frequency and severity—with time. Scheduling miltefosine after a meal alleviated GI symptoms for some patients. Importantly, miltefosine-induced diarrhea and vomiting should receive special attention, as dehydration may further increase the risk of renal failure.

Urogenital system and teratogenicity: Miltefosine has been shown to be teratogenic and embryotoxic in animal models, even in sub-therapeutic concentrations [32]. Its use is strictly contraindicated during pregnancy, and because of its long half-life in plasma, contraceptives are advised for 5 months after treatment for women of child-bearing age [33].

Van Thiel et al. were the first to report, in 2010, that miltefosine may cause the diminution (and even complete absence) of ejaculate, as well as other symptoms, including decreased libido, scrotal tenderness, and epididymitis [22]. In this study, male urogenital system disorders were common, with 39% of the male patients reporting one or more reproductive system disorders, including orchialgia, decreased volume of ejaculate, erectile disorders, inability to orgasm, and decreased libido. Testicular pain was, in some cases, severe and necessitated the discontinuation of treatment. Epididymitis was diagnosed via ultrasound in three patients who suffered severe scrotal pain, suggesting that its prevalence may be underestimated. Male urogenital symptoms typically occurred in the last 2 weeks of treatment. All cases resolved after the discontinuation of miltefosine and the initiation of treatment with antibiotics and NSAIDs.

Despite numerous clinical studies having been conducted, AEs related to the male reproductive system are only rarely mentioned in the literature. Our results suggest that these AEs are very common but are under-recognized and under-reported as patients tend not to report embarrassing issues unless specifically asked [22]. A study from Bangladesh supported the high prevalence of disturbances in the male reproductive system, as 9 out of 29 male patients demonstrated abnormal semen analysis findings during treatment with miltefosine [34].

The cutaneous AEs observed in our study can be categorized into those directly related to the CL lesions or those representing other skin manifestations. The suppuration of existing CL lesions and the development of inflamed subcutaneous nodules were documented in 17.6% of patients. These findings have only been rarely described with respect to miltefosine treatment [22,35]. An increased immune response to the parasitic debris released during anti-leishmanial treatment may possibly explain this clinical observation.

Other cutaneous AEs observed in our study included the exacerbation of acne vulgaris and folliculitis. The co-occurrence of acne exacerbation and an inflamed pilonidal cyst, observed in a young female patient, may suggest a possible effect of miltefosine on the follicular unit. Bullous eruption and generalized pruritus developed in two patients but rapidly resolved after discontinuation of miltefosine. Although cutaneous AEs have not been frequently described in the previous literature, we believe that the fact that patients in our study were examined in dermatological clinics may have contributed to a higher awareness of cutaneous AEs during treatment.

Musculoskeletal AEs: Six patients reported a sudden onset of severe chest and/or back pain, being of a pleuritic nature in some. Symptoms resolved in all cases after the discontinuation of treatment. In addition, aseptic monoarthritis of the knee and unilateral sacroiliitis were documented in two patients. Arthritis during miltefosine treatment has been described, but it is exceedingly rare. Interestingly, gout exacerbation was recently reported under miltefosine treatment [27].

Renal impairment with a transient, mild increase in serum creatinine during treatment is a well-described AE of miltefosine. However, in our cohort, increased creatinine levels and acute renal failure were observed in 20% of the patients. This potentially serious AE typically occurred during the last two weeks of treatment. The concomitant use of nephrotoxic drugs and the presence of vomiting and diarrhea, which may lead to pre-renal azotemia, should be evaluated as potential risk factors for the development of renal impairment. It should be noted that monitoring the CRP level during treatment may be of benefit, as elevated levels heralded the development of renal failure in some cases.

Although miltefosine has been used against leishmaniasis for three decades, new discoveries and insights regarding its safety continue to emerge. The World Health Organization recently published a statement regarding measures to minimize the risk of ocular adverse events with miltefosine based on the work of the Advisory Committee on Safety of Medicinal Products (ACSoMP). A recently published review on this topic described eight studies involving 31 leishmaniasis patients who were treated with miltefosine and developed ocular complications. Among these cases, uveitis, keratitis, scleritis, and Mooren’s ulcer led to vision impairment and even blindness [36]. Ocular complications developed after miltefosine treatment for an average duration of 47 days. In our study, the patients did not undergo routine ophthalmic examination. Fortunately, no cases of acute ophthalmic complications were observed.

The global medical and economic impact of leishmaniasis is significant. According to recent WHO estimates, up to one million new cases of leishmaniasis occur annually across more than 100 countries. This highlights an urgent, unmet need for new strategies to fight the global burden of leishmaniasis, ideally through a combination of environmental prevention, new treatments, and, most importantly, the development of a safe and effective vaccine. Currently, none of the available therapeutic alternatives for leishmaniasis can be considered a gold standard as they all have limited efficacy, multiple toxicities, contraindications, and considerable cost. Until a vaccine is developed, innovative applications of current treatments could enhance our antileishmanial armamentarium and optimize the benefits of existing medications. In this context, topical formulations of miltefosine, including liposomal versions, may offer improved penetration and efficacy while reducing the risk of systemic AEs [37].

## 5. Limitation

A limitation of this study is the relatively limited sample size of patients. Despite this fact, our results increase the body of knowledge regarding known and previously unreported toxicities of miltefosine, adding to the growing data on this subject.

## 6. Conclusions

Miltefosine is a principal component in the therapeutic arsenal against visceral and cutaneous leishmaniasis. Our study demonstrated that the tolerability for miltefosine is low and that its use may be associated with severe toxicities. More studies are needed to better define the role of miltefosine for the treatment of CL based on its risk–benefit ratio.

## Figures and Tables

**Table 1:** Characteristics of patients and leishmania species. Epidemiological characteristics of 68 patients with cutaneous leishmaniasis. Leishmania species were identified for all patients by polymerase chain reaction–restriction fragment length polymorphism (PCR-RFLP). Statistical significance difference between genders was found in terms of body weight (*p*-value = 0.0002).

Characteristics of Patients	Total	Males	Females
Number of Patients	68	54	14
Age, mean (SD)	30.3 (15.6)	29.1 (15.5)	35 (15.8)
[median, range]—years	[23, 18–88]	[23, 18–88]	[32, 19–65]
Weight (SD)	75.8 (13.8)	78.5 (13.5)	66.4 (11)
[median, range]—Kg	[72, 54–120]	[75, 62–120]	[63, 54–99]
***Leishmania* Species**			
*L. major* (%)	37 (54.4)	28 (51.9)	9 (64.3)
*L. tropica* (%)	12 (17.6)	8 (14.8)	4 (28.6)
*L. braziliensis* (%)	18 (26.4)	17 (31.5)	1 (7.1)
*L. infantum* (%)	1 (1.5)	1	0

**Table 2:** Tolerability, treatment course outcome, and the severity of adverse events (AEs) during miltefosine treatment for CL. No statistically significant differences were found between genders.

	Total, N (%)	Males, N (%)	Females, N (%)
	(Out of 68)	(Out of 53)	(Out of 15)
Treatment completed	44 (64.7)	36 (67.9)	8 (53.3)
Required temporary discontinuation of treatment or dose modification	4 (5.9)	2 (3.8)	2 (13.3)
Required permanent discontinuation of treatment	20 (29.4)	15 (28.3)	5 (33)
Referred to hospitalization	7 (10.3)	6 (11.3)	1 (6.6)
Free of any AEs	6 (8.8)	5 (9.4)	1 (6.6)
Mild AEs	29 (42.6)	23 (43.4)	6(40)
Moderate AEs	16 (23.5)	13 (24.5)	3 (20)
Severe AEs	17 (25)	13 (24.5)	4 (26.6)

**Table 3:** Adverse events and laboratory abnormalities were evaluated at initiation of miltefosine treatment and every 1–2 weeks thereafter. No statistically significant differences were found between genders.

	Total, N (%)	Males, N (%)	Females, N (%)
	(Out of 68)	(Out of 53)	(Out of 15)
Any GI AEs	45 (66.1)	36 (69.9%)	9 (60%)
Nausea and vomiting	38 (55.8)	30 (56.6)	8 (53.3)
Abdominal pain	33(48.5)	25 (47.1)	8 (53.3)
Diarrhea	22 (32.3)	16 (30.1)	6 (40)
Malaise	16 (23.5)	11 (19.6)	5 (33.3)
Suppuration of cutaneous lesions (including local or regional dissemination)	12 (17.6)	11 (19.6)	1 (6.6)
Acne exacerbation	8 (11.8)	6 (11.3)	2 (13.3)
Severe generalized musculoskeletal pain	6 (7.6)	5 (9.4)	1 (6.6)
URTI-like	6 (8.8)	4 (7.5)	2 (1.3)
Arthritis	2 (2.9)	2 (3.8)	0 (0)
Rash	1 (1.5)	0	1 (6.6)
Pruritus	1 (1.5)	1 (1.9)	-
**Male reproductive system disorders**			
AEs related to male genital organ disorder—any	NA	21 (39.6%)	NA
Orchialgia	NA	15 (28.3%)	NA
Decreased volume of ejaculate	NA	11 (20.8%)	NA
Erectile dysfunction	NA	5 (9.4%)	NA
Inability to orgasm	NA	5 (9.4%)	NA
Decreased libido	NA	5 (9.4%)	NA
**Laboratory abnormalities**			
Elevated creatinine level	14 (20.6%)	11 (20.8%)	3 (20%)
Elevated LFT	12 (17.6%)	10 (18.9%)	2 (13.3%)
Anemia	3 (4.4.%)	3 (5.7%)	0 (0%)
Neutropenia	1 (1.5%)	1 (1.9%)	0 (0%)
Thrombocytopenia	1 (1.5%)	1 (1.9%)	0 (0%)
Hyperkalemia	1 (1.5%)	1 (1.9%)	0 (0%)
Leukocytosis	1 (1.5%)	1 (1/9%)	0

**Table 4:** AEs leading to permanent (A) or temporary (B) treatment discontinuation or dose reduction. No statistically significant differences were found between genders.

	Total, N (%)	Males, N (%)	Females, N (%)
	(Out of 68)	(Out of 53)	(Out of 15)
**A. Causes of early discontinuation of treatment**			
Severe renal failure	8 (11.7)	6 (11.8)	2 (13.3)
Orchialgia, epididymitis	5 (7.4)	5 (9.4)	NA
Severe GI symptoms	3 (4.4)	1 (5.7)	2 (13.3)
Severe, acute, musculoskeletal pain	3 (4.4)	3 (5.6)	0
Bullous eruption	1 (1.5)	0	1 (6.7)
Suppuration/worsening of existing lesions	2(2.9)	2 (3.8)	0
Hyperkalemia	1 (1.5)	1 (1.9)	0
Arthritis	1 (1.5)	1 (1.9)	0
Total	24 (35.3%)	19 (35.8)	5 (33.3)
**B. Causes of temporary discontinuation or dose reduction**			
GI symptoms	2 (2.9)	0	2 (13.3)
Orchialgia	1 (1.5)	1 (1.9)	NA
Elevated LFT	1 (1.5)	1 (1.9	0
Total	4 (5.9)	2 (3.8)	2 (13.3)

## Data Availability

Study data are available from the corresponding author upon request.
